# Comparison between SBR Compounds Filled with In-Situ and Ex-Situ Silanized Silica

**DOI:** 10.3390/polym13020281

**Published:** 2021-01-16

**Authors:** Pilar Bernal-Ortega, Rafal Anyszka, Yoshihiro Morishita, Raffaele di Ronza, Anke Blume

**Affiliations:** 1 Department of Mechanics of Solids, Surfaces & Systems (MS3), Chair of Elastomer Technology & Engineering, Faculty of Engineering Technology, University of Twente, 7500 AE Enschede, The Netherlands; m.d.p.bernalortega@utwente.nl (P.B.-O.); r.p.anyszka@utwente.nl (R.A.); 2 Bridgestone EU NV/SA, Italian Branch–Technical Center, Via del Fosso del Salceto, 00128 Rome, Italy; yoshihiro.morishita@bridgestone.com (Y.M.); Raffaele.DIRONZA@bridgestone.eu (R.d.R.)

**Keywords:** silica, silanes, silanization, rubber

## Abstract

The main advantages of the use of silica instead of carbon black in rubber compounds are based on the use of a silane coupling agent. The use of a coupling agent to modify the silica surface improves the compatibility between the silica and the rubber. There are two different possibilities of modifying the silica surface by silane: ex-situ and in-situ. The present work studies the differences between these processes and how they affect the in-rubber properties of silica filled SBR compounds.

## 1. Introduction 

Nowadays, silica has become along with carbon black, the most important filler in the rubber industry. The use of silica instead of carbon black in passenger car tire tread compounds leads to a lower rolling resistance, equal wear resistance and higher wet grip [1,2,3]. However, the presence of silanol groups at the silica surface causes a polar surface of the silica particles. This polar nature promotes the filler-filler interactions by the formation of hydrogen bonds. Therefore, big non-dispersed silica clusters are created inside the unipolar rubber matrix. To avoid these strong filler-filler interactions and improve the compatibility with the rubber it is necessary to modify the silica surface. For this purpose, bi-functional organo-silanes such as bis (triethoxysilylpropyl) tetrasulfide (TESPT) or bis(triethoxysilylpropyl) disulfide (TESPD) are commonly used [4,5].

Usually, the modification of the silica surface using silane coupling agents is performed during the mixing process. The silica, the organosilane and the rubber are mixed at once in an internal mixer. During this procedure two chemical reactions take place: (i) bonding of the organosilane to the silica surface and (ii) reaction between the silane and the rubber. This mixing method exhibits some disadvantages, such as the need for an additional step to ensure the finalization of the reaction between the silane and the silica and the generation of ethanol as a by-product of the silanization reaction [6,7,8]. The first one leads to longer mixing times, the second contains the risk of porosity at the surface of the extrudate.

To overcome these disadvantages, one of the possible solutions could be the pre-silanization of the silica. This process consists of the surface modification of the silica with the coupling agent in a chemical reactor which takes place before the mixing. This pre-modification of the filler avoids the additional mixing step and the treatment of the release of ethanol during the mixing process [8]. Furthermore, it might lead to higher yields of silanization than the ones of the in-situ process due to the missing complex rubber matrix during the chemical reaction, by avoiding side reactions. If this conclusion is valid the final properties of the rubber compounds could be improved in terms of lower viscosity, higher reinforcement or a decrease in the Payne effect. 

In order to analyze the effect of the different silanization processes on the in-rubber properties, different compounds prepared using in-situ and ex-situ silanization were evaluated and compared in the present work. The influence of the different silanization processes was studied by the analysis of Mooney viscosity, Payne effect, crosslink density and mechanical properties. The results were obtained from compounds filled with unmodified, in-situ and ex-situ silanized silica. 

In this work, two different types of silanes were used, a bi-functional and a mono-functional silane. In the rubber field, the terms “bi-functional” and “mono-functional” referring to silanes, are related to their reactivity towards the silica and the rubber. Bi-functional silanes present a dual reactivity. The organic functional part can react with the rubber matrix while the alkoxy groups can react with the silica surface. However, mono-functional silanes only react with the silica surface [4,9]. For this study, TESPD was used as coupling agent and hexadecyltrimethoxysilane as a covering agent (CA). TESPD is a bi-functional silane that can couple to the silica surface as well as to the polymer. However, the CA, is a mono-functional silane that can only couple to the silica surface. It can be used in the rubber industry as an additional silane to decrease the filler-filler network even further. Normally, the ethoxy-version is used to avoid the release of methanol with the known disadvantage of a slow reaction time. 

A pre-modified silica might have the disadvantage that unmodified silica surface will be exposed during the mixing process due to the break of the silica clusters into smaller units. To evaluate this possible effect, additional TESPD was added during the mixing process to the compound filled with pre-modified silica. The same was done by replacing the additionally added TESPD by the covering agent.

## 2. Materials and Methods

### 2.1. Materials 

The rubber used in this work was non-functionalized SSBR Buna VSL 4058 (Lanxess, Cologne, Germany). The selected silica was ULTRASIL 7000 GR (Evonik Industries, Wesseling, Germany). Bis(triethoxysilylpropyl) disulfide (TESPD) (Evonik, Wesseling, Germany) was used as silane coupling agent and hexadecyltrimethoxysilane (Millipore Sigma, Hamburg, Germany) as covering agent (CA). For the preparation of the rubber compounds Zinc oxide (ZnO) and stearic acid were used as activators (Millipore Sigma, Hamburg, Germany); sulfur and *Ν*-tert-butyl-benzothiazole sulfonamide (TBBS) (Caldic B.V., Rotterdam, The Netherlands) as curatives and treated distillate aromatic extracted (TDAE) (Hansen & Rosenthal, Hamburg, Germany) as oil. 

### 2.2. Pre-Silanization of Silica

The modification process of the silica was the same for both modifying agents, TESPD and CA. The procedure was performed in one single step using toluene as the reaction medium. The silica and the modifying agent were mixed together in toluene and then heated up to 80 °C for 24 h. Afterwards the sample was filtered and dried in an oven to eliminate all the solvent. 

### 2.3. Compounding and Mixing 

Rubber compounds were prepared in an internal mixer (Brabender Plasticorder 350S, Duisburg, Germany) with a fill factor of 0.7, an initial temperature of 100 °C and a rotor speed of 50 rpm. For the present study the following samples were prepared, according to the formulation shown in Table 1 and the mixing procedure in Table 2:

R1: filled with unmodified silica and without silane

R2: in-situ silanized during mixing with TESPD

R3: filled with unmodified silica and addition of covering agent during mixing

R4: pre-silanized silica with TESPD 

R5: pre-silanized silica with covering agent

R6: pre-silanized silica with TESPD + addition of TESPD during mixing 

R7: pre-silanized silica with TESPD + addition of covering agent during mixing. 

R8: pre-silanized silica with covering agent + addition of covering agent during mixing. 

## 3. In-Rubber Tests

### 3.1. Cure Behavior

The vulcanization process was studied by a Rubber Process Analyzer, RPA 2000 from Alpha Technologies (Hudson, OH, USA), at 160 °C by applying a deformation of 6.98% at a frequency of 1.667 Hz. The *t*_90_, that is, the time to reach 90% conversion, of each compound was used as molding time for vulcanizing the rubber samples in a hydraulic press.

### 3.2. Mooney Viscosity

The Mooney viscosity (ML (1 + 4), 100 °C) was analyzed using a Mooney viscosimeter (MV 2000VS, Alpha Technologies, Hudson, OH, USA) according to ASTM D1646. 

### 3.3. Payne Effect

A study of the Payne effect was performed by a Rubber Process Analyzer, RPA 2000 from Alpha Technologies (Hudson, OH, USA) with strain sweeps from 0.56% to 100% for uncured samples, a frequency of 1.6 Hz and a temperature of 60 °C. 

### 3.4. Crosslink Density

The crosslink density of the studied compounds was determined by two different methods: equilibrium swelling and solvent freezing point depression.

**Figure 1:**
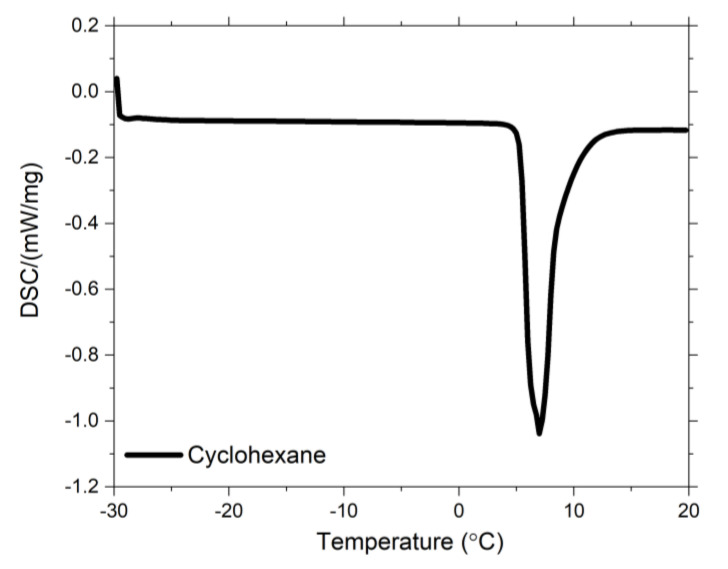
DSC freezing curve for pure cyclohexane.

- The crosslink density by equilibrium swelling experiments was obtained using the Flory-Rehner equation [10]. Five vulcanized samples of each compound were swollen in toluene at room temperature for a period of 7 days. The samples were previously extracted with acetone in order to remove the oil and unreacted cure agents.
- The determination of the freezing point depression temperature was carried out using a Differential Scanning Calorimetry DSC 214 Polyma, from Netzsch-Gerätebau (Selb, Germany). Small pieces of the vulcanized rubber (previously acetone-extracted), around 3 mm^2^, were swollen in cyclohexane for 3 days to reach the equilibrium swelling. The swollen samples were then placed into DSC pans with an excess of cyclohexane to ensure that the solvent is trapped inside the polymer network. Cyclohexane was used as solvent because it shows a clear crystallization peak in DSC (Figure 1) and it is a good swelling solvent for SBR [11,12,13]. The cooling/heating rate was 5 °C/min under nitrogen atmosphere. The freezing point depression of solvents imbibed in swollen vulcanizates has been studied by many authors [12,13,14,15,16,17]. These studies showed that the magnitude of the depression of the solvent in a swollen vulcanizate is related to the degree of crosslink density. By comparison of the transition temperature values of a confined and a free solvent, a freezing point depression temperature (Δ*T_f_*) can be calculated as the difference between the freezing temperature of pure cyclohexane (Figure 1) and that of the solvent that is trapped in the rubber compounds. This parameter depends on the mesh size of the polymer network. When more crosslinks connect the polymer chains, the mesh size becomes smaller, the solvent is confined in a tighter network constraining the crystallization of the solvent [12]. The higher the crosslink density the lower the swelling. This means that less solvent is trapped in the polymer resulting in a lower intensity of the peak. The freezing point of the solvent is the sum of the freezing point of the “free” solvent and that of the “trapped” solvent. If less solvent is trapped, this freezing temperature becomes lower which means that the freezing point depression is larger. The larger the freezing point depression the higher the crosslink density.

### 3.5. Macro Dispersion

The macro dispersion of the filler in the rubber compound was studied by means of a Dispergrader—Dispersion Tester Alpha View (Alpha Technologies, Hudson, OH, USA). The rubber samples were investigated by an optical light microscopy with 30° irradiation angle and at 100× magnification. 

### 3.6. Tensile Test

The tensile strength of the cured samples was measured by a universal testing machine Zwick Z05 (Zwick, Ulm, Germany) operation with the crosshead speed of 500 mm/min according to ASTM D412 standard.

## 4. Results

### 4.1. Pre-Silanization of Silica

**Figure 2:**
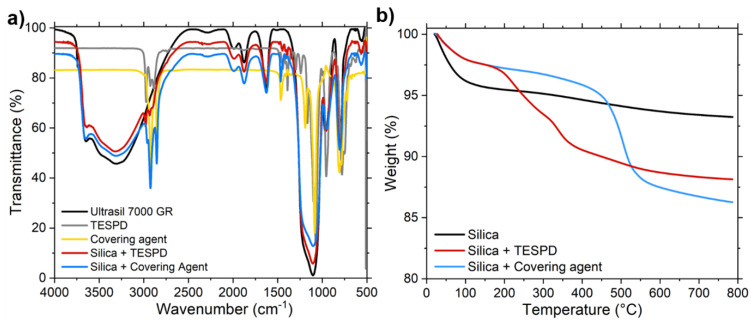
(**a**) Fourier Transform Infrared Spectroscopy (FTIR) analysis of the of the unmodified and modified silicas with bis(triethoxysilylpropyl) disulfide (TESPD) (red line) and covering agent (blue line), TESPD (grey line) and covering agent (CA) (yellow line) and (**b**) thermogravimetric analysis (TGA) curves of the unmodified and modified silicas with TEPSD (red line) and covering agent (blue line).

The success of the functionalization process was analyzed by Fourier Transform Infrared Spectroscopy (FTIR) (Perkin Elmer, Waltham, MA, USA) using the DRIFTS (diffuse reflectance infrared Fourier transform spectrometry) cell. This technique is commonly used to analyze samples than can be ground into a fine powder, that is, silica. The modification was confirmed for both samples by the presence of the band at a ~2965 cm^−1^, corresponding to the symmetric and asymmetric stretching of –CH_2_ groups and the band at ~1480 cm^−1^, assigned to –CH_3_ groups (Figure 2a).

The quantification of the amount of silane that was attached to the silica was performed using Thermogravimetric Analysis (TGA) using a TA 550 device (TA Instruments, New Castle, DE, USA) operating under an air atmosphere with a heating rate of 20 °C/min from room temperature to 800 °C. The quantity of modifying agents at the surface of the silica was approximately 9% for the TESPD and 10% for the covering agent (Figure 2b).

### 4.2. In-Rubber Tests

#### 4.2.1. Mooney Viscosity

**Figure 3:**
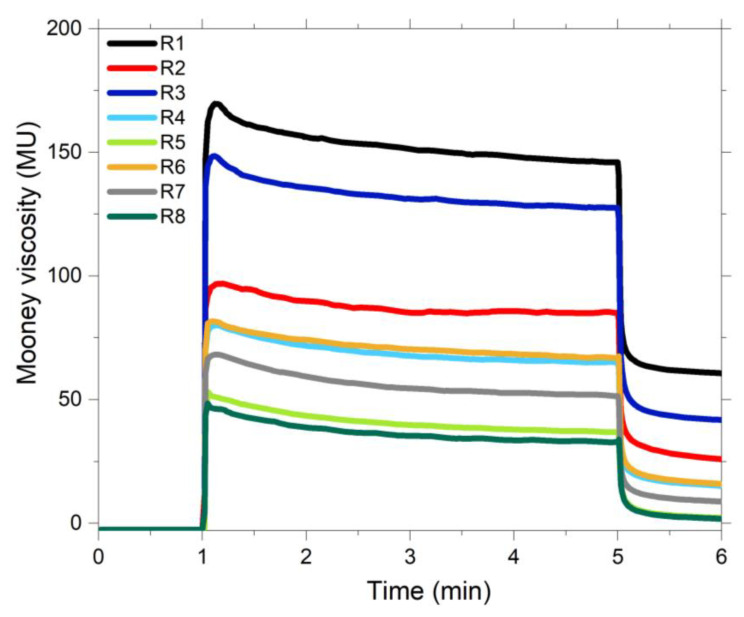
Mooney viscosity of the studied samples.

The Mooney viscosity of the studied compounds is depicted in Figure 3. It can be observed that the lowest values are obtained with the samples pre-modified with the covering agent (R5 and R8). The mono-functional nature of the covering agent and its lower polarity compared to TESPD, results in better hydrophobation of the silica. This leads to lower filler-filler interactions which results in a lower viscosity of the rubber compounds. The results of the samples with TESPD (R2, R4, R6 and R7) show that the ones in which the silica was pre-modified the Mooney viscosity is significantly lower compared to the in-situ silanized samples. This could indicate that the pre-silanization was more effective than the reaction of TESPD during the two-stage mixing process. It is important to note that it is most likely that the silanization reaction with TESPD is not finished during the mixing process and that for the full silanization the curing step is required as well. 

Finally, the samples R1 (unmodified silica) and R3 (unmodified silica with addition of CA during mixing) present the highest values of viscosity. For the sample R1, this behavior is caused by the absence of silane in this compound, the polar nature of the unmodified silica promotes the formation of a strong filler-filler network. In R3, in which 2 phr of CA were added during the mixing process, the viscosity decreases in comparison to R1. The addition of a small amount of the monofunctional silane reduces the filler network and therefore the viscosity of this sample compared to R1. The rate of the reaction of the CA is much lower than that of TESPD, therefore the coupling efficiency in the given mixing time is much lower for the CA [18]. For this reason, R3 presents higher Mooney viscosity than the sample which is in-situ silanized with TESPD (compound R2). It can be expected that the silanization reaction continues during the vulcanization.

#### 4.2.2. Payne Effect

**Figure 4:**
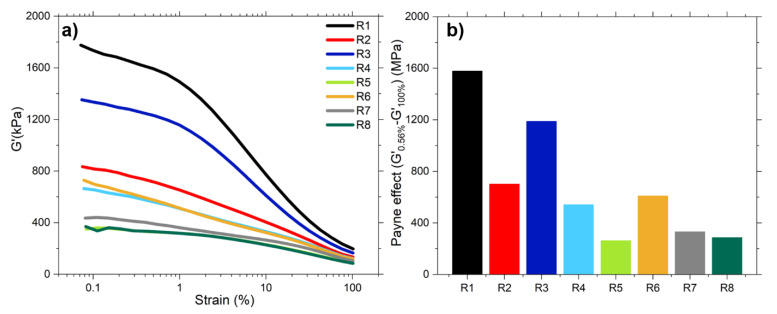
(**a**) Uncured Payne effect and (**b**) ΔG’ of the studied samples.

The Payne effect of the studied samples was analyzed for uncured samples (Figure 4). It is the difference between the storage moduli at 0.56% and 100% of strain. The lowest Payne effect was obtained for the pre-modified samples with covering agent (R5 and R8). The main explanation for these results is the much better shielding effect of the long alkyl chain of the CA compared to the TESPD. For this reason, in these compounds the filler-filler interactions are reduced and therefore the Payne effect is lower. Comparing the samples in-situ and pre-modified with TESPD (R2, R4, R6 and R7), it can be observed that the ones with the pre-modified silica present a lower Payne effect than the in-situ silanized sample (R2). As mentioned above, this could be an indication that the ex-situ silanization was more effective than the in-situ silanization with TESPD in the given mixing time. It is required to find the best compromise between the improvement of the silanization and the risk of overcuring. It was already investigated by Luginsland [19] that the optimum mixing conditions for TESPD are in the range of a dump temperature of 145–155 °C. Additionally, the in-situ silanization could be improved by adding a third mixing stage to ensure the finalization of the reaction between the silane and the silica.

The compound R7 presents a similar Payne effect than the samples R5 and R8. The addition of a covering agent during mixing R7, reduces the silica clusters during the compounding process. For pre-modified silica, new unmodified surface can be uncovered during the mixing process which might causes the formation of a more pronounced filler-network. When additional silane is added to this freshly created silica surface, it can cover this surface and avoid by this the formation of stronger filler-filler interactions. In the sample R6, with the addition of TESPD during mixing, the Payne effect is higher compared to R7. As explained above, the longer alkyl chain of the CA has a higher shielding effect than TESPD reducing to a higher degree the interaction between the filler particles. 

Finally, the samples R1 and R3, present a much higher Payne effect than the other compounds. Regarding R1, the absence of a coupling or covering agent in the sample leads to the formation of a strong filler network. For R3, the addition of a small quantity of CA during the mixing process reduces the Payne effect compared to R1 but due to the insufficient coupling reaction it is still significantly higher than the obtained ones for the other compounds. 

#### 4.2.3. Vulcanization

**Figure 5:**
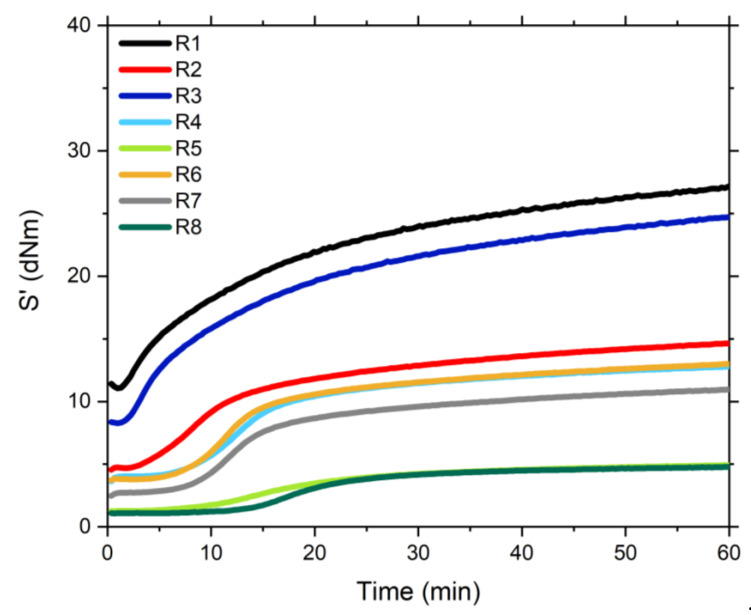
Vulcanization curves of the studied samples.

The vulcanization curves of the different silica/SBR compounds are shown in Figure 5. The cure curves of the samples R1 and R3 show a pronounced torque rise at the beginning of the vulcanization process. These behavior is associated with the flocculation of the silica, that is, re-clustering of the silica particles after heating the compounds [20]. This effect is reduced in the samples containing TESPD and suppressed for the samples pre-modified with the covering agent. The modification of the surface of the silica strongly reduces the filler-filler interactions and hinders the re-clustering. Furthermore, the cure curve for the sample filled with unmodified silica (R1) indicates that the pristine silica has a slower vulcanization process than the samples with modified silica. This phenomenon has been widely studied and it is related to the surface chemistry of silica. The presence of acidic groups such as silanol and siloxane which absorb basic accelerators, decelerates the vulcanization process [21,22].

It also can be observed that the samples R5 and R8 show a very low maximum torque. The final torque is caused by the polymer-polymer network, the filler-rubber and the filler-filler interactions. In these cases, the filler network is strongly reduced due to the presence of the covering agent which cannot couple to the rubber matrix. Therefore, a really low maximum torque is obtained. During the vulcanization the silanization reaction seems to be completed, which results in the successful shielding of the silica surface.

#### 4.2.4. Crosslink Density 

**Figure 6:**
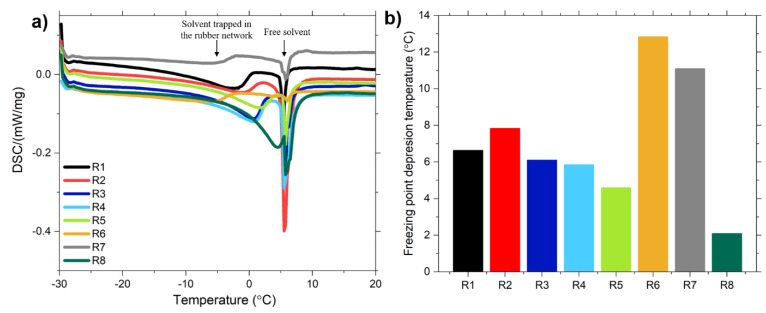
(**a**) DSC freezing curves for the swollen compounds and (**b**) Freezing point depression of the compounds.

The DSC freezing curves and the freezing point depression of the swollen rubber compounds are shown in Figure 6. In all DSC curves two differentiate peaks can be observed: the peak of the free solvent and the peak of the solvent trapped in the rubber network. The depression of the freezing temperature of the solvent is clearly visible for all samples. On the one hand, the samples that present the largest freezing point depression are R6 and R7, both pre-modified with TESPD and with the addition during mixing of TESPD (R6) and CA (R7). The intensity of the peak of the trapped solvent is lower in these samples. This is explained by a smaller mesh size due to a higher crosslink density of these compounds due to the covalent bonds formed between the silica and the rubber because of the presence of TESPD that reacts with the rubber matrix. The highest crosslink density is obtained for the compound R6. As mentioned above, the addition of TESPD during the mixing process can cover the new freshly created silica surface and couple to the polymer creating more covalent bonds in this compound. For the compound R7, the addition of the covering agent during mixing covers the new silica surface created but does not couple to the rubber due to the mono-functional nature of the CA. The presence of additional TESPD and CA shields the silica surface more effectively, hindering the absorption of accelerators and ZnO and leading to an increase of their active concentration and therefore a higher crosslink density is obtained. It has to be noted that the sample R1 (unmodified silica) shows a relatively high freezing depression point. The high freezing depression point can be explained by the presence of a mechanical or physical three-dimensional mesh formed by the strong filler-filler interactions [20]. This mesh restricts the swelling, leading to a higher freezing depression point which assumes a higher value of crosslink density for this compound. Regarding the samples which are pre-modified with covering agent (R5 and R8), they present the lowest values of the freezing depression point. These results can be explained by the absence of a filler network and polymer-filler coupling.

**Figure 7:**
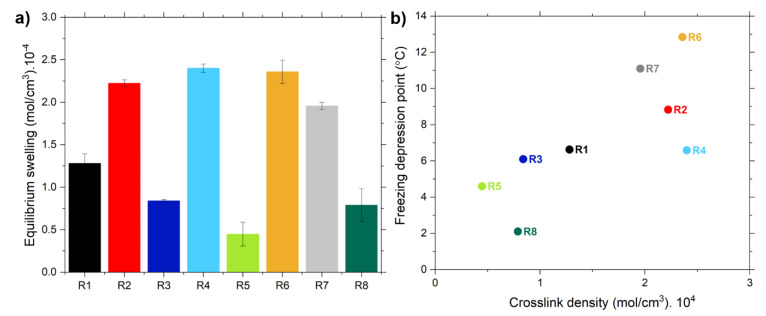
(**a**) Crosslink density measured by equilibrium swelling and (**b**) correlation between the crosslink density results obtained by freezing point depression and equilibrium swelling.

Analyzing the crosslink density of the compounds measured by equilibrium swelling (Figure 7a) experiments, it can be observed that all samples containing modified silica with TESPD have a similar crosslink density. The manner of modifying the silica (in-situ or ex-situ) does not have a great impact on the rubber network. In these samples the crosslink density is much higher than in the ones with unmodified silica or modified with covering agent. The covalent bonds formed between the silica and the rubber due to the presence of TESPD increase the overall crosslink density. However, for the sample R7, in which the covering agent is added during the mixing process the crosslink density is slightly inferior. A possible explanation for this result could be that the silanization reaction is not completed after the mixing process and the coupling of TESPD competes with the reaction of the CA during the curing process, resulting in a non-effective coupling from the silica to the polymer. The other possible explanation is that the CA reduces further the filler-filler network, leading to a lower crosslink density. 

These results correlate (Figure 7b) with the ones obtained with the determination of the freezing point depression. Both techniques show that the samples containing TESPD are the ones with higher crosslink density. However, some differences can be observed, with the values of the Δ*T_f_*, indicating that the samples in which the silica was pre-modified have a higher crosslink density. This could be related to the differences among the two different methods, such as the solvent (cyclohexane and toluene) or the days of swelling of the samples (3 days for the freezing point depression and 7 days for equilibrium swelling). In fact the swelling degree increases with the increasing time of the experiment, which results in lower values of crosslink densities for longer times. 

#### 4.2.5. Macro-Dispersion 

**Figure 8:**
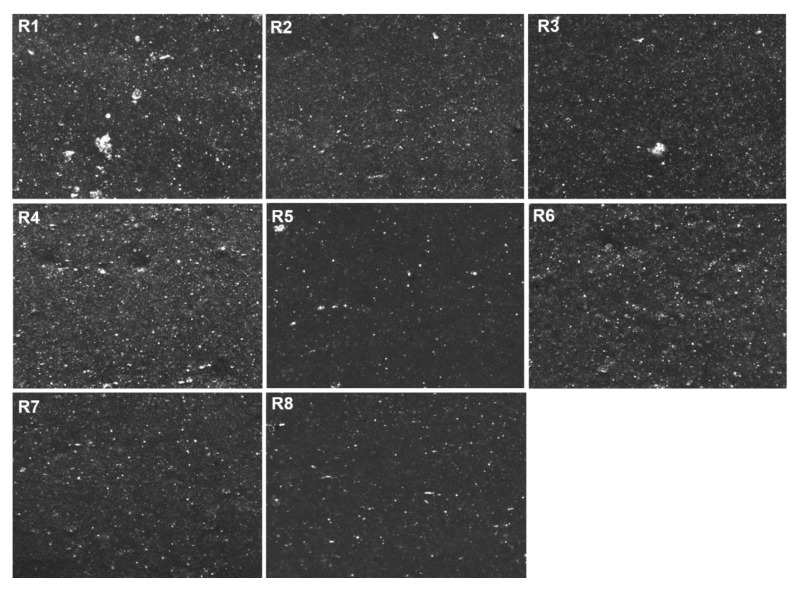
Dispergrader images of the SBR/silica compounds.

The macro-dispersion of the silica particles in the rubber matrix was analyzed by optical microscopy. The images and data results obtained for each rubber sample are shown in Figure 8 and Table 3. The images and data presented in Figure 8 and Table 3 show that all samples that contain silane in the formulation (TESPD or covering agent) show a good overall macro-dispersion despite the high filler concentration. However, some differences can be observed. The samples with covering agent (R3, R5 and R8) show a better macro-dispersion than the samples containing TESPD, due to the lower filler filler-interactions in these cured compounds. The samples R5 and R8 present the higher values of dispersion and the lower average cluster size. Regarding the samples silanized with TESPD (in-situ or ex-situ), R7 shows the best dispersion and lower average cluster size, which can be explained by the on-top addition of the covering agent during the mixing process. As already mentioned above, the covering agent covers the freshly created silica surface during mixing, avoiding the formation of filler-filler interactions and therefore the formation of clusters. Finally, the sample with unmodified silica (R1) shows the worst macro-dispersion of all compounds, big clusters can be observed. This result was expected due to the high filler-filler network caused by the absence of silane in this compound. 

#### 4.2.6. Mechanical Properties

**Figure 9:**
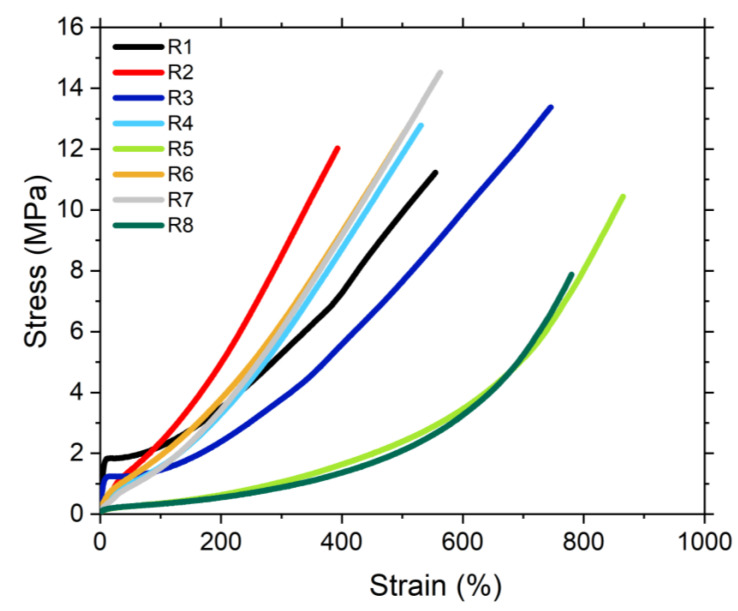
Stress-strain curves of the studied compounds.

The mechanical properties of the studied compounds were analyzed by their performance in a tensile test (Figure 9). These properties are the result of different molecular mechanisms (polymer network, polymer-filler interactions, hydrodynamic effect and filler-filler interactions) [23].

In Table 4 the tensile strength, elongation at break and modulus at 100% and 300% of the studied compounds are shown. The reinforcement level of the silica in the studied samples is the result of different factors. For the compound filled with non-modified silica (R1) this reinforcement is associated to the strong filler-filler network, while in the samples containing TESPD in the formulation (R2, R4, R6 and R7) this reinforcement is the result of the formation of covalent bonds between the silica and the rubber. Finally, in the case of the samples with covering agent (R3, R5 and R8), the decrease in the mechanical properties is caused by the suppression of the filler-filler interactions and missing filler-polymer coupling. These samples have the highest elongation at break but lowest M300, showing the effect of a well-dispersed silica but without any coupling to the rubber matrix. By the use of the mono-functional coupling agent the compatibility of the filler to the polymer matrix increases, which improves the rheological, curing and mechanical properties [24].

As can be observed in Figure 9, the samples R1 and R3 show a shoulder at the beginning of the stress-strain curve, which results from the strong filler-filler network in this samples. This strong filler network leads to a high stiffness of these compounds, resulting in higher values of the modulus at very low strains. In these compounds, the rubber trapped in the filler network behaves as a filler in terms of stress-strain properties increasing the modulus. With increased stress and strain the filler clusters break, releasing the rubber trapped in the filler network which is visible by a kink in the curves. 

**Figure 10:**
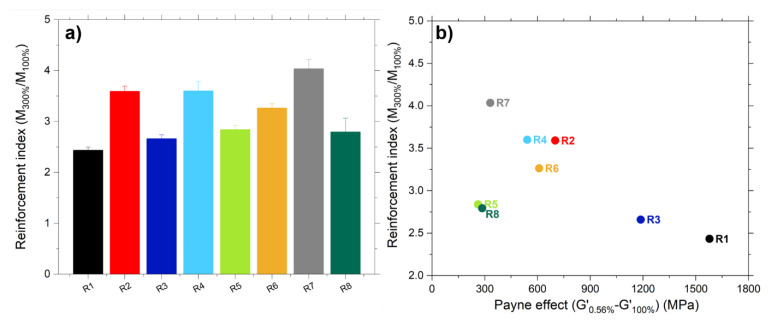
(**a**) Reinforcement index of the studied samples and (**b**) Correlation between the reinforcement index and the Payne effect of the studied compounds.

The reinforcement index (calculated as the ratio between the modulus at 300% strain and the modulus at 100% strain) of the studied samples is shown in Figure 10a. The samples containing TESPD present the highest values. As previously discussed, the addition of the coupling agent and the consequent formation of covalent bonds between the silica and the rubber, improves the interaction of the filler with the matrix and as result the reinforcing behavior is improved in these compounds. The compound R7 shows the highest reinforcement, the additional addition of CA during the mixing covers the newly created silica surface and reduces the filler-filler interactions. As a result, a better dispersion of the filler was obtained, resulting in improved mechanical properties. In the case of R6, with addition of TESPD during mixing, the shielding of the silica surface is less effective than with the CA and therefore the reinforcement is lower in this compound compared to R7.

In Figure 10b, it can be observed that there is an inverse correlation between the reinforcement index and the Payne effect. The Payne effect can be used to study the filler status, that is, the micro-dispersion of the filler in the rubber. Therefore, a better micro-dispersion of the filler in the matrix would lead to a lower Payne effect [7]. Observing Figure 10b, it can be concluded that the dispersion of the silica has an enormous effect on the mechanical properties of the rubber compounds. On the one hand, the sample R1 (unmodified silica) has the lowest reinforcing effect and the highest Payne effect, because of the low compatibility of the silica with the rubber due to its polar nature. On the other hand, R7 (pre-modified with TESPD and addition of CA during mixing) shows the highest reinforcement index and the lowest Payne effect. The addition of the CA during compounding, with a long alkyl chain, has a shielding effect of the freshly created silica. This allows to reduce the interaction between particles and therefore achieving a better dispersion and consequently improved mechanical properties. 

Regarding the samples R5 and R8, it can be observed that they are outside the correlation between the reinforcement index and the Payne effect. The pre-modification of the silica only with the covering agent, reduces in a higher degree the Payne effect compared to the other compounds. However, the mono-functional nature of the covering agent and the consequent missing polymer-filler interaction combined with the low filler-filler network leads to poor mechanical properties. 

## 5. Conclusions

This work presents experimental results that show the potential of the pre-silanization of the silica surface as an alternative to the in-situ silanization during mixing of rubber compounds. The study performed on SBR/silica compounds revealed that the method of modifying the silica surface has a great effect on the final properties of the compounds. The samples in which the silica was pre-modified in a chemical reactor, present lower Mooney viscosity, lower Payne effect and similar mechanical properties and crosslink density than the ones in-situ silanized. These results could indicate that the pre-silanization is more effective than the in-situ silanization during a two stage mixing. It has to be noted, that a two stage mixing is a short mixing cycle for the performance of an in-situ silanization. The silanization reaction could not be completed during this mixing process and for the further silanization the curing step is required. Although the silanization reaction is finalized in the curing step, the in-rubber properties of this compounds will be sufficient and comparable with the ones obtained for the ex-situ silanization. Therefore, by accepting the no optimized results in the green compound, the final performance of the in-situ modified and only 2-stages mixed compounds is sufficient.

The combination of using pre-modified silica with the addition of covering agent or TESPD during the mixing process (compounds R7 and R6) have shown the best performance in the in-rubber properties. As previously explained, during the mixing process of compounds using pre-modified silica as the filler, new unmodified surface can be generated. The formation of this new unmodified silica surface could lead to the formation of big clusters and subsequently to the formation of a strong filler-filler network. By incorporation of additional covering agent during mixing, this silane can cover the new unmodified surface. In this work, the comparison between the addition of TESPD (compound R6) and a covering agent (compound R7) during the mixing process has shown that the covering agent has a greater effect on the in-rubber properties. The addition of the mono-functional silane in R7, with a long alkyl chain, covered in a higher degree the new generated silica surface compared to R6. As a consequence, in this sample the particle-particle interactions are decreased, obtaining a lower Payne effect and a better dispersion in the rubber matrix. As a consequence of the better dispersion of the silica, this compound also presents the best performance in terms of mechanical properties and crosslink density.

## Figures and Tables

**Table 1:** Formulation of the rubber compounds in phr.

Compounds	SBR (phr)	Silica * (phr)	TESPD * (phr)	Covering Agent * (phr)	TDAE (phr)	Other Ingredients (phr)
R1	100	80	-	-	37.5	Zinc Oxide-2.5 Stearic Acid-2.5 Sulphur-1.4 TBBS-2
R2	100	80	6.2	-	37.5	
R3	100	80	-	2	37.5	
R4	100	87.2	-	-	37.5	
R5	100	89.5	-	-	37.5	
R6	100	87.2	2	-	37.5	
R7	100	87.2	-	2	37.5	
R8	100	89.5	-	2	37.5	

(*) In the samples R4, R5, R6, R7 and R8, the TESPD or the covering agent were already bonded to the silica. The quantity of the modifying agent bonded to the silica was determined by thermogravimetric analysis (TGA).

**Table 2:** Mixing procedure of the rubber compounds.

Time (min)	Action
**Step 1** pre-heating 100 °C-50 rpm	
0.00	Addition of rubber, mastication
1.20	Addition of 1/3 filler, 1/2 silane (TESPD or covering agent)
2.40	Addition of 1/3 filler, 1/2 silane, (TESPD or covering agent), TDAE
4.00	Addition of 1/3 filler, Zinc Oxide, Stearic Acid
5.00	Increase of the torque (increase temperature to 130 °C)
10.00	Stop mixing (reaching 140 °C)
**Step 2** pre-heating 50 °C-50 rpm	
0.00	Addition elastomer pre-mix, mastication
1.30	Addition curatives (sulphur, TBBS)
3.00	Stop mixing

**Table 3:** Dispersion and average cluster size of the studied compounds.

Compound	Dispersion (%)	Average Cluster Size (µm)
R1	70	3.21
R2	75	2.84
R3	83	2.67
R4	79	2.82
R5	87	2.23
R6	81	2.78
R7	85	2.69
R8	88	2.32

**Table 4:** Mechanical properties of the studied compounds.

Compound	Ts, MPa	Eb, %	M100	M300
R1	11.1 ± 1.5	535 ± 44	2.2 ± 0.10	5.4 ± 0.4
R2	11.4 ± 1.7	385 ± 40	2.2 ± 0.10	8.0 ± 0.4
R3	12.9 ± 1.0	730 ± 43	1.4 ± 0.10	3.7 ± 0.1
R4	12.7 ± 0.7	525 ± 32	1.6 ± 0.20	5.8 ± 0.5
R5	9.2 ± 1.1	840 ± 36	0.4 ± 0.01	1.1 ± 0.02
R6	11.7 ± 0.2	480 ± 25	1.9 ± 0.05	6.3 ± 0.3
R7	13.4 ± 0.7	530 ± 26	1.5 ± 0.05	6.1 ± 0.4
R8	8.1 ± 0.5	770 ± 40	0.3 ± 0.01	0.9 ± 0.1

## Data Availability

The data presented in this study are available on request from the corresponding author. The data are not publicly available due to privacy restrictions.
